# Survival Benefit of First Single-Organ Deceased Donor Kidney Transplantation Compared With Long-term Dialysis Across Ages in Transplant-Eligible Patients With Kidney Failure

**DOI:** 10.1001/jamanetworkopen.2022.34971

**Published:** 2022-10-07

**Authors:** Susanne Strohmaier, Christine Wallisch, Michael Kammer, Angelika Geroldinger, Georg Heinze, Rainer Oberbauer, Maria C. Haller

**Affiliations:** 1 Center for Public Health, Department of Epidemiology, Medical University of Vienna, Vienna, Austria; 2 Center for Medical Statistics, Informatics, and Intelligent Systems, Section for Clinical Biometrics, Medical University of Vienna, Vienna, Austria; 3 Division of Nephrology and Dialysis, Department of Medicine III, Medical University of Vienna, Vienna, Austria; 4 Ordensklinikum Linz, Elisabethinen Hospital, Department of Medicine III, Nephrology, Hypertension, Transplantation, Rheumatology, Geriatrics, Linz, Austria

## Abstract

**Importance:**

Kidney transplant is considered beneficial in terms of survival compared with continued dialysis for patients with kidney failure. However, randomized clinical trials are infeasible, and available evidence from cohort studies is at high risk of bias.

**Objective:**

To compare restricted mean survival times (RMSTs) between patients who underwent transplant and patients continuing dialysis across transplant candidate ages and depending on waiting time, applying target trial emulation methods.

**Design, Setting, and Participants:**

In this retrospective cohort study, patients aged 18 years or older appearing on the wait list for their first single-organ deceased donor kidney transplant between January 1, 2000, and December 31, 2018, in Austria were evaluated. Available data were obtained from the Austrian Dialysis and Transplant Registry and Eurotransplant and included repeated updates on wait-listing status and relevant covariates. Data were analyzed between August 1, 2019, and December 23, 2021.

**Exposures:**

A target trial was emulated in which patients were randomized to either receive the transplant immediately (treatment group) or to continue dialysis and never receive a transplant (control group) at each time an organ became available.

**Main Outcomes and Measures:**

The primary outcome was time from transplant allocation to death. Effect sizes in terms of RMSTs were obtained using a sequential Cox approach.

**Results:**

Among the 4445 included patients (2974 men [66.9%]; mean [SD] age, 52.2 [13.2] years), transplant was associated with increased survival time across all considered ages compared with continuing dialysis and remaining on the wait list within a 10-year follow-up. The estimated RMST differences were 0.57 years (95% CI, –0.14 to 1.84 years) at age 20 years, 3.01 years (95% CI, 2.50 to 3.54 years) at age 60 years, and 2.48 years (95% CI, 1.88 to 3.04 years) at age 70 years. The survival benefit for patients who underwent transplant across ages was independent of waiting time.

**Conclusions and Relevance:**

The findings of this study suggest that kidney transplant prolongs the survival time of persons with kidney failure across all candidate ages and waiting times.

## Key Points

**Question:**

What is the difference in 10-year restricted mean survival time when receiving a kidney transplant versus remaining on the wait list and continuing dialysis for transplant-eligible patients with kidney failure across different ages?

**Findings:**

In this cohort study including 4445 adults on the wait list for their first single-organ deceased donor kidney transplant, application of target trial emulation methods showed a difference in terms of restricted mean survival times. Kidney transplant prolonged life expectancy across patient ages compared with continuing dialysis.

**Meaning:**

The findings of this study suggest that transplant-eligible patients with kidney failure should not be excluded from transplant programs based on their age.

## Teaser

This cohort study uses target trial emulation to examine survival time in patients with kidney failure who undergo kidney transplant vs those who receive dialysis across ages and waiting time to transplant.

## Introduction

Kidney transplant is considered the optimal treatment strategy for eligible patients with kidney failure. Numerous studies have shown that transplant is cost-effective and improves survival compared with long-term dialysis.^1,2,3,4,5^ However, this body of evidence consisting of observational studies does not allow for causal interpretation.

Well-designed and well-conducted randomized clinical trials constitute the standard for establishing causality. Randomized treatment allocation to kidney replacement therapy, however, is not feasible. Hence, quantifying the survival benefit achieved from kidney transplant in terms of differences in 10-year restricted mean survival times (RMSTs) must rely on observational studies.

In attempts to mitigate bias in causal estimates from observational research, the concept of target trial emulation was introduced.^6^ A target trial is an idealized randomized clinical trial that is not feasible and is therefore mimicked through the application of design principles from randomized clinical trials to the analysis of observational data.^7^

Given the scarcity of robust evidence to underpin the anticipated survival advantage for kidney transplant recipients, it remains unclear whether kidney transplant improves survival for patients eligible for transplant of all ages and whether the duration of pretransplant dialysis reduces anticipated survival benefits for transplant recipients compared with their peers remaining on a wait list while receiving long-term dialysis.^8,9,10^

Our hypotheses were that survival benefits achieved with kidney transplant diminish with increasing recipient age and as waiting time with maintenance dialysis lengthens owing to an increasing frailty of the patient population. The primary aim of our study therefore was to investigate survival benefit between kidney transplant and long-term dialysis for transplant-eligible patients across different candidate ages. Our secondary aim was to investigate survival benefit depending on waiting time with dialysis across ages of transplant candidates.

## Methods

### Study Design and Data Source

We conducted a retrospective cohort study that emulated a target trial evaluation approach described by Hernán and Robins^6^ using the Austrian (Oesterreichische) Dialysis and Transplant Registry (OEDTR), which was established in 1965 by the Austrian Society of Nephrology and has almost complete follow-up data. The contained information was thoroughly extracted from medical records originally documented by the responsible physician at the follow-up visits. The study was approved by the Ethics Committee of the Medical University Vienna, performed in accordance with the Declaration of Helsinki,^11^ and reported in accordance with the Strengthening the Reporting of Observational Studies in Epidemiology (STROBE) reporting guideline.^12^ Patients consented to data collection in the OEDTR at the time of initiation of kidney replacement therapy.

We retrieved data from the OEDTR on demographic characteristics (sex, age, weight, height [information on race and ethnicity are not specified in the database, and most of the Austrian population is White]), type of kidney disease (vascular nephropathy, hereditary kidney disease, glomerulonephritis, and diabetic nephropathy), comorbidities (diabetes, coronary heart disease, myocardial infarction or instable angina pectoris, congestive heart failure, other heart disease, neoplasia, liver disease, cerebrovascular disease, peripheral vascular disease, and chronic obstructive pulmonary disease), and number of blood pressure–lowering drugs. The data were supplemented by information from the Eurotransplant database, including the wait-listing status (transplantable, nontransplantable, high urgent, immunized kidney, and highly immunized kidney), the first wait-listing date, transplant date, and death date. Information on various patient characteristics, comorbidities, blood pressure–lowering medication, and wait-listing status were updated approximately yearly throughout the follow-up period.

We included all transplant-eligible patients who started receiving dialysis between January 1, 2000, and December 31, 2018. Transplant eligibility was determined by wait-listing status after January 1, 2000. Patients were followed up until their date of death, loss to follow-up, or end of follow-up on June 30, 2019, whichever came first. We included all transplants from deceased donors occurring between January 1, 2000, and the end of follow-up. We excluded patients younger than 18 years at the time of first wait-listing; patients registered for multiorgan transplant; those with implausible dates regarding wait-listing status, death, or transplant; and patients with missing measurements at first wait-listing. Because only a minimal proportion of values were missing in the follow-up recordings, we decided to use last observation carried forward or backward, assuming that comorbidities (eg, coronary artery disease) do not recede. More details are available in eMethods 2 in the Supplement.

### Statistical Analysis

The characteristics of patients at the time of their first wait-listing are described by means (SDs) for continuous variables and frequencies and percentages for categorical variables. Data analysis was conducted between August 1, 2019, and December 23, 2021.

We developed a protocol for a hypothetical target trial that applies design principles from randomized clinical trials to the analysis of observational data. The target trial was then emulated using the observed data mimicking patient selection by applying appropriate inclusion criteria, randomization by confounder adjustment, and treatment adherence by adjustments for informative censoring. Herein, we describe the target estimand and the corresponding estimation procedure. eMethods 1 in the Supplement provides further details.

#### Target Estimand

The primary end point of interest in our study was the time from transplant allocation to death from any cause. Particularly, we aimed to compare the 2 treatment strategies—receive a transplant from a nonliving donor vs remain waitlisted while undergoing dialysis and never receive a transplant—across different ages and quantify the contrast in terms of differences in RMSTs. The RMST has the interpretation of a mean survival time of a population within a prespecified time horizon; hence, differences in RMST indicate gain or loss in the mean survival time within the specified period.^13^ Secondarily, we were interested in differences in RMST conditional on different durations of wait-listing before treatment allocation. To allow comparison with the literature, we additionally report the results of our main analysis in terms of survival curves and hazard ratios.

#### Estimation Procedure

To assess the target estimand, we followed a sequential Cox approach^14,15^ creating a series of auxiliary trials. Auxiliary trials started each time a transplant occurred (ie, a transplant was allocated). For each auxiliary trial, the trial population consisted of patients actively waitlisted who had survived for at least the same amount of time since being waitlisted. At each transplant allocation time, patients who underwent transplant were classified as treated, and patients who continued dialysis were classified as controls. Patients in the control group who underwent transplant during follow-up of an auxiliary trial were artificially censored at the time of transplant because their treatment did not adhere to the assigned strategy from this point onward (remain on wait list and never undergo transplant). These patients were subsequently assigned to the treated group in a later auxiliary trial of the series. Data from all auxiliary trials were stacked into a single data set, and the outcome models to address the main research questions were applied to this data set.

To deal with confounding and nonadherence to the assigned treatment strategy, we estimated stabilized inverse probability of treatment weights (IPTWs) as well as annual stabilized inverse probability of censoring weights (IPCWs).^16^ Inverse probability of treatment weights were estimated for each patient at each transplant allocation time based on a multivariable logistic regression model including sex, the year of first appearance on the wait list, dialysis use before wait-listing (yes/no), and the most recent updates of age, body mass index, comorbidities (diabetes, coronary heart disease, myocardial infarction or instable angina pectoris, congestive heart failure, other heart disease, history of neoplasia, liver disease, cerebrovascular disease, and peripheral vascular disease), number of blood pressure–lowering medications, time from wait-listing to transplant allocation, and episodes of nontransplantable status before transplant allocation (yes/no) as covariates, which were selected based on the common cause criterion.^17^

The annual stabilized IPCWs for the control groups were estimated using the same covariates in separate Cox models per auxiliary trial. In the treated groups, the IPCWs were set to 1. Final weights were computed as the product of stabilized IPTWs and IPCWs, and subsequently winsorized at the 0.1st and 99.9th percentile of their distribution. Different cutoffs were applied in sensitivity analyses.

We fit a weighted Cox proportional hazards model for all-cause survival, with our main exposure of interest (treatment allocation) as the only independent variable. By incorporating the final weights, the Cox model was dynamically adjusted for measured confounders selected based on the common cause criterion and corrected for treatment nonadherence (ie, later transplant in the control cohort).

To address the primary aim of our study and evaluate whether the differences in RMSTs achieved by transplant vary by age at transplant allocation, we fit a Cox model including interaction terms between treatment and age. Age was flexibly modeled using a restricted cubic spline with knots at the 1st, 25th, 50th, 75th, and 99th percentiles of the age distribution.^18^

To investigate our secondary aim of whether the difference in RMSTs achieved by transplant depended on the time spent on wait list before transplant allocation we estimated separate models among patients who had been on the wait list up to 1 year, between 1 and 2 years, or more than 2 years. We included interaction terms for treatment and age as described above in all 3 models.

From the fitted Cox models, we computed the marginal differences in restricted mean survival times^13^ comparing transplant with remaining on the wait list. Effect size estimates are reported with 95% CIs based on the 2.5th and 97.5th percentiles from the distribution of 1000 bootstrap resamples of all patients.

Data preparation and analyses were conducted using R statistical software, version 4.0.1 (R Foundation for Statistical Computing). Further information about data management, handling of missing data, and details on the analyses steps outlined in this section can be found in eMethods 2 in the Supplement.

## Results

**Figure 1.**
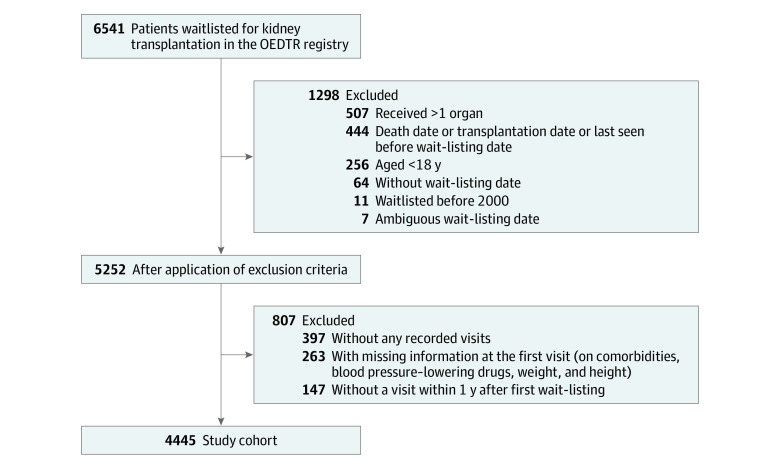
Derivation of the Study Cohort OEDTR indicates Austrian (Oesterreichische) Dialysis and Transplant Registry.

The study cohort comprised 4445 patients who met the eligibility criteria (Figure 1). The population included 1471 women (33.1%) and 2974 men (66.9%); mean (SD) age was 52.2 (13.2) years (Table). The most frequent underlying primary kidney disease was glomerulonephritis (1235 [27.8%]) followed by diabetic nephropathy (777 [17.5%]), hereditary kidney disease (720 [16.2%]), and vascular nephropathy (698 [15.7%]).

**Table.** Characteristics of the Study Cohort at the Time of First Appearance on Wait List

Characteristic	No. (%)
No.	4445
Sex	
Female	1471 (33.1)
Male	2974 (66.9)
Age, mean (SD), y	52.2 (13.2)
BMI, mean (SD)	26.6 (5.0)
Kidney disease etiology	
Diabetic nephropathy	777 (17.5)
Glomerulonephritis	1235 (27.8)
Hereditary	720 (16.2)
Vascular	698 (15.7)
Other	1015 (22.8)
Dialysis before wait list placement	4237 (95.3)
If so, time on dialysis before wait-listing, mean (SD), d	478.9 (505.2)
BP-lowering drugs, mean (SD), No.	2.6 (1.5)
Comorbidities	
Diabetes	1061 (23.9)
Coronary heart disease	671 (15.1)
Myocardial infarction or instable angina pectoris	177 (4.0)
Congestive heart failure	307 (6.9)
Other heart disease	561 (12.6)
History of neoplasia	279 (6.3)
Liver disease	360 (8.1)
Cerebrovascular disease	456 (10.3)
Peripheral vascular disease	553 (12.4)

Abbreviations: BMI, body mass index (calculated as weight in kilograms divided by height in meters squared); BP, blood pressure.

In total, 3621 of 4445 patients (81.5%) received a kidney transplant and 1392 patients (31.3%) died. Transplants were performed between September 16, 2000, and May 23, 2019. The median time from wait-listing to kidney transplant was 1.7 years, from wait-listing to date-last-seen was 8.0 years, and from wait-listing to death was 13.4 years. The median time from beginning of dialysis to kidney transplant was 3.2 years. The follow-up was not long enough to determine the IQRs.

Target trial emulation resulted in 1367 auxiliary trials, comprising a total of 2 067 620 person-trials.^15^ Characteristics of the study population at treatment allocation of the auxiliary trials can be found in eTable 1 in the Supplement. The percentage of person-trials that were artificially censored owing to transplant during the follow-up was 80.9% (1 673 203 person-trials). Missing information on one or more comorbidities, number of blood pressure–lowering drugs, height, or weight was encountered in 2.6% of the observations.

### Kidney Transplant and All-Cause Mortality

Our analysis based on target trial emulation revealed a marginal increase in RMST for patients who underwent transplant compared with those who remained on the wait list for 0.53 years (95% CI, 0.45-0.63 years) over a follow-up of 5 years and an increase of 2.40 years (95% CI, 2.09-2.95 years) over a follow-up of 10 years. eFigure 1A in the Supplement shows RMST curves for patients who underwent transplant and those remaining on the wait list and continuing dialysis, as well as differences in RMST. eFigure 1B in the Supplement displays the marginal effect in terms of survival curves and differences in survival probabilities over time.

### Kidney Transplant and All-Cause Mortality Across Different Ages

**Figure 2.**
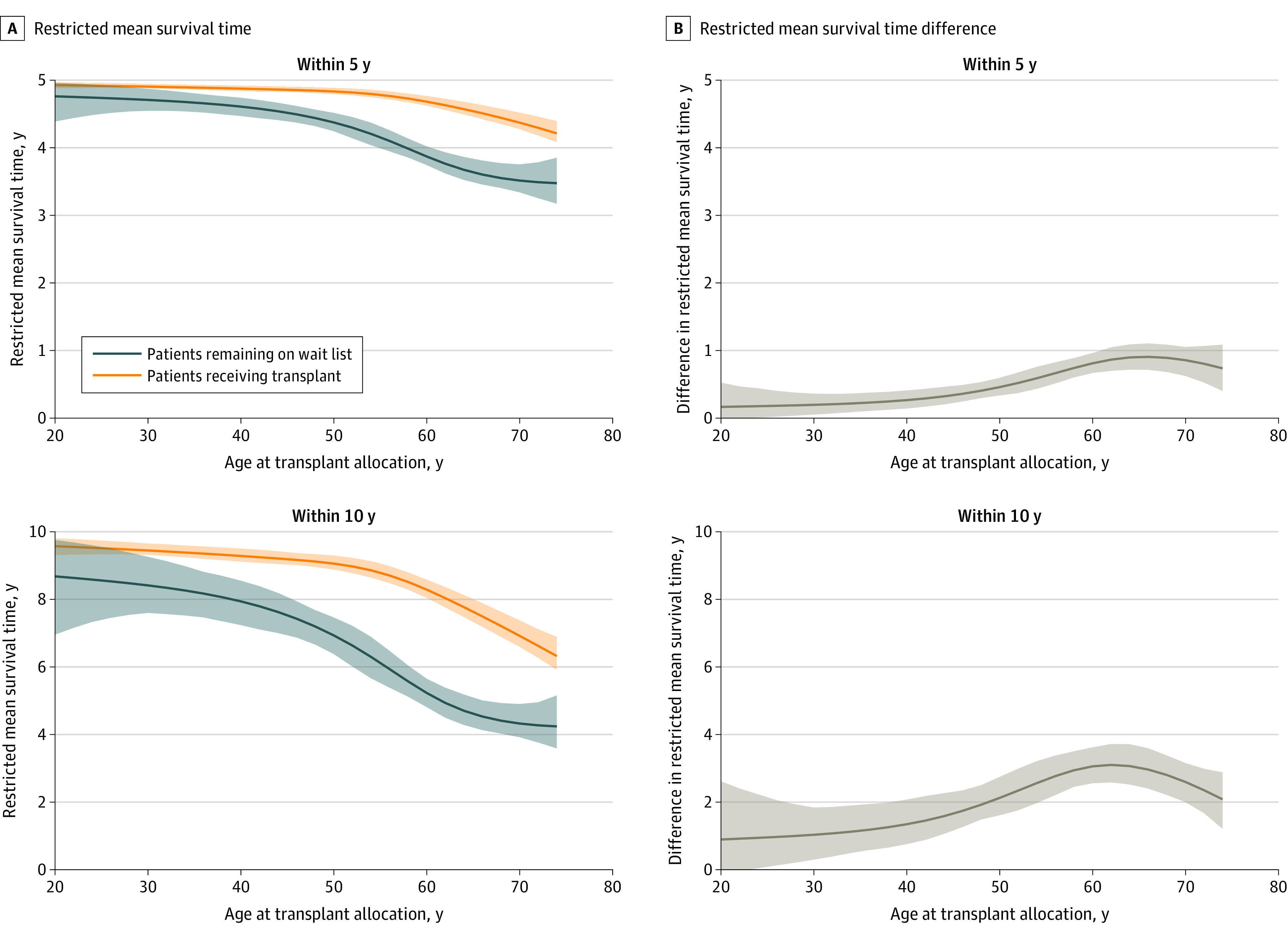
Restricted Mean Survival Times for All-Cause Mortality and Differences Thereof A, Five-year and 10-year restricted mean survival times for all-cause mortality. B, Five-year and 10-year restricted mean survival times for all-cause mortality differences. Shaded areas indicate 95% CIs.

Figure 2 shows that, for patients aged 20 years, the 95% CIs for the RMST differences included 0: the 5-year RMST difference was 0.10 (95% CI, –0.02 to 0.33) and the 10-year RMST difference was 0.57 (95% CI, –0.14 to 1.84). The mean life-years gained within 5 years of follow-up increased with age, for example, the 5-year RMST difference for age 50 years was 0.41 (95% CI, 0.31-0.52) and for 60 years was 0.77 (95% CI, 0.63-0.91). For the 10-year RMSTs, we observed a similar behavior until age 60 years, for example, the 10-year RMST difference for patients aged 50 years was 2.03 (95% CI, 1.56-2.55) and for those aged 60 years, the gain was 3.01 (95% CI, 2.50-3.54). For patients aged 70 years, however, the gain in mean survival time was less pronounced, with an RMST difference of 2.48 (95% CI, 1.88-3.04). eTable 2 in the Supplement lists RMST differences for varying ages and time horizons of follow-up.

### Kidney Transplant and All-Cause Mortality by Wait List Time

**Figure 3.**
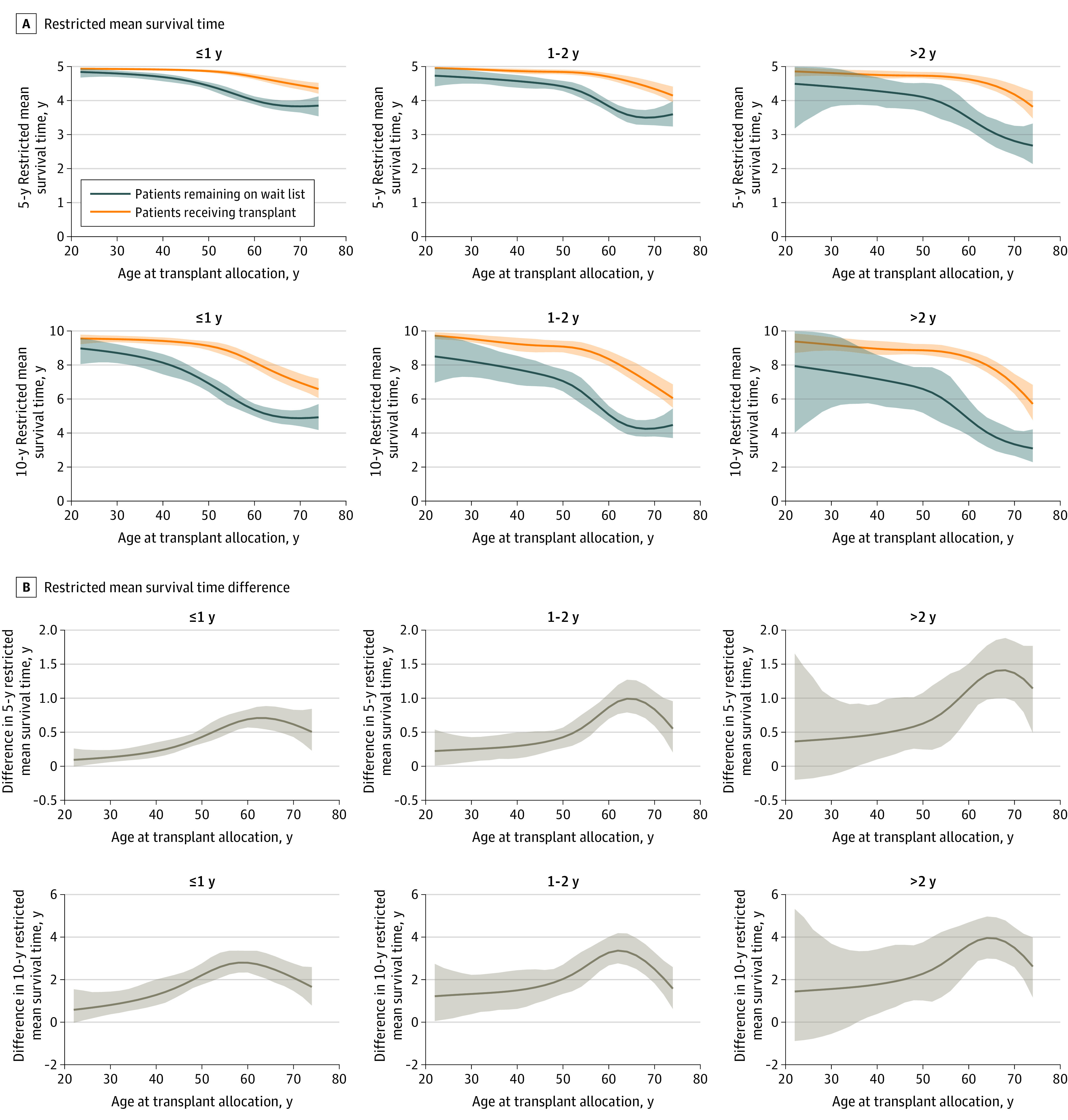
Restricted Mean Survival Times Conditional on Wait-listing Duration A, Five-year and 10-year restricted mean survival times for all-cause mortality conditional on different times on wait list. B, Five-year and 10-year restricted mean survival times for all-cause mortality and differences conditional on different times on wait list. Shaded areas indicate 95% CIs.

Figure 3 shows the 5-year and 10-year RMST differences for varying ages conditional on wait list durations (≤1, 1-2, or >2 years). These stratified analyses revealed a similar pattern as described in the previous paragraph. Again, the differences in 5-year and 10-year RMSTs were more pronounced for patients aged 55 to 65 years and smaller for older patients. The largest RMST differences were obtained in the analysis conditional on having been on the wait list for longer than 2 years, but the corresponding 95% CIs were wide and included 0 for patients younger than 35 years.

### Alternative Effect Size Scales

To allow comparison with the previous studies, we report estimated survival curves (eFigure 2 and eFigure 3 in the Supplement) and estimated hazard ratios (eFigure 4 and eFigure 5 in the Supplement). Expressed as hazard ratio, the marginal effect size was 0.28 (95% CI, 0.19-0.33).

We again observed wide 95% CIs, including 1 on the hazard ratio scale (eFigure 4 in the Supplement) for patients aged 20 years. Similarly, the 95% CIs in the stratified analyses were wider, especially for younger patients when conditioned on having been on the wait list for 2 or more years (eFigure 5 in the Supplement).

### Sensitivity Analyses

eFigure 6 in the Supplement shows the distribution of the final stabilized and winsorized weights used for the main analyses. Standardized differences comparing the covariates included in the IPTW models between patients who did vs did not undergo transplant before and after IPTW are presented in eTable 3 in the Supplement. The largest absolute standardized difference before IPTW was 0.398, observed for the binary indicator of noneligibility periods before the start of the auxiliary trials. In contrast, after IPTW, the absolute standardized difference was smaller than 0.027 for all covariates. In sensitivity analyses, we applied different winsorizing cutoffs for the final weights. In addition, we considered unstabilized IPTWs and IPCWs and calculated IPCWs for 6-month intervals instead of annual intervals. None of the considered alternatives changed our conclusions, as illustrated by eFigure 7 in the Supplement showing the 10-year RMST conditional on age for patients who underwent vs did not undergo transplant.

## Discussion

Our study based on target trial emulation provides evidence for longer life expectancy after kidney transplant compared with long-term dialysis across different transplant candidate ages and irrespective of time on wait list. We observed a greater survival benefit among older patients who had survived for more than 2 years on the wait list. This finding can likely be explained by the increased mortality rate among older patients continuing to receive dialysis.

A large systematic review published in 2011 concluded that kidney transplant was associated with reduced mortality, but findings were at high risk for bias.^4^ Various methodologic flaws contributed to a likely overestimation of the benefits of kidney transplant. Many of the included studies had issues, such as (1) important confounders were not adjusted for dynamically throughout follow-up, ignoring the development of illness while patients were on the wait list; (2) periods of temporary ineligibility for transplant were not taken into account; (3) outcomes of transplant recipients were compared with those of patients continuing dialysis ineligible for transplant; (4) outcomes of transplant recipients after graft failure were attributed to dialysis; (5) survival time until transplant was artificially guaranteed for those who eventually received a transplant vs patients who continued dialysis may have died at any time during follow-up; or (6) kidney transplant was modeled as a time-dependent variable using standard survival methods shown to be inferior when the efficacy of an intervention is evaluated in the presence of time-dependent confounding by dynamic treatment allocation.^19^

Since the aforementioned systematic review was published,^4^ other studies reported advantageous survival for kidney transplant recipients.^20,21^ In a national registry study from Denmark, kidney transplant was associated with survival benefit despite severe comorbidities and older age.^21^ However, the study did not take temporary withdrawal from the wait list into account and comorbidities were not updated dynamically. Another northern European registry analysis found a very large survival advantage of almost 14 years for kidney transplant recipients compared with patients on the wait list who were receiving dialysis, classifying patients into treatment groups at the start of kidney replacement therapy.^20^ Classifying individuals as transplant recipients at the start of kidney replacement therapy, however, introduces immortal time bias.^22^

A recent French registry study reported a 6.8-month gain in life expectancy for patients who undergo transplant compared with transplant-eligible patients receiving dialysis at 10-year follow-up.^23^ This study applied an interesting approach to trial emulation with propensity score–matched comparison groups. The chosen approach makes results for the marginal effect size comparable to the reports described in our study. However, in our study, patients in the control group were required to never undergo transplant, but the French registry study allowed for later transplant in the control group. This variation could in part explain any differences in the marginal effect estimates. Unlike the French registry study, in addition to estimating marginal effects, we investigated the survival benefit across transplant candidate ages as well as conditional on different durations patients receiving dialysis were on the wait list before transplant allocation.

Multiple studies reported worsening of survival in kidney transplant recipients as dialysis vintage accumulates.^9,24,25,26^ These studies, however, described outcomes among patients who actually received a kidney transplant and compared dialysis vintage characteristics, but do not allow conclusions about the effectiveness of transplant in those who are eligible and may receive a transplant at a particular dialysis vintage. We believe our results noting greater survival benefit with increased dialysis vintage is a signal that cohorts of transplant-eligible patients receiving dialysis change over time. During the first 2 years of dialysis, transplant-eligible patients are well enough to survive while receiving dialysis, which results in a smaller net benefit from transplant. As dialysis vintage accumulates, mortality increases, resulting in a greater survival benefit of transplant (because mortality would be higher if dialysis was continued). Our study does not contradict knowledge about increasing mortality rates as dialysis vintage accumulates, but rather is, to our knowledge, the first study to use target trial emulation methods to quantify the survival benefit of kidney transplant in terms of restricted mean survival time in patients who are eligible to receive such a treatment given a particular dialysis vintage.

### Strengths and Limitations

Strengths of our approach include a clearly defined target estimand and accompanying target trial protocol that defines time 0 for the outcome estimand of interest. Building a series of auxiliary trials allowed us to dynamically adjust for repeatedly measured confounders and ensured the inclusion of only patients eligible for transplant, thus taking the development of disease and temporal withdrawal from the wait list into account. In addition, considering only patients who survived equally long while on the wait list at the start of an auxiliary trial further mitigated the risk of immortal time bias.

The study has limitations. Although the long-term follow-up with a reported observation period of 10 years is a strength of our registry data, it is difficult to determine the survival benefit of kidney transplant in younger patients with kidney failure who have a life expectancy longer than 10 years. Therefore, the findings showing only a small survival benefit in younger patients should not be interpreted as ineffectiveness of kidney transplant, but rather as a limitation of follow-up in this subgroup. Furthermore, the relatively small sample size, resulting in wide 95% CIs, implies some caution is needed when interpreting the results for younger patients.

Deriving estimates from target trial emulation methods involves strong structural assumptions. Specifically, we assumed that we had sufficient information on confounders to ensure exchangeability of our treatment groups after adjusting by means of IPTWs. The OEDTR is a closely monitored national registry with a negligible number of patients lost to follow-up and long-term data periodically updated information by the responsible physician. The registry offered high-quality data with information on a rich set of potential confounders that were incorporated by a careful covariate selection and modeling approach. Nevertheless, as for any observational study, there is still a risk for bias owing to unmeasured confounding. Furthermore, we assumed that each participant had a positive probability to receive either treatment at each transplant allocation time (positivity assumption). Because we only considered patients on the wait list and hence eligible for transplant, we can rule out any structural violation of the positivity assumption. Any sampling-related violations would have been detected during the inverse probability of treatment weight calculations, for example, by running into divergence or separation problems.

In addition, our approach based on sequential Cox models implies the proportional hazards assumption, but it is well known that hazards are nonproportional shortly after transplant because the mortality rate within the first year after transplant is higher than the rate for patients undergoing dialysis. However, the main focus of our study was long-term life expectancy after kidney transplant when the differences from early follow-up are mitigated. Furthermore, missing data in the registry were a concern. Because only 2.6% of the observations were incomplete, we decided to use last observation carried forward or backward, assuming that comorbidities (eg, coronary artery disease) do not recede.

Our study population is representative for a Central European, primarily White, population, and our findings may not be generalizable to populations in other regions of the world or with different racial and ethnic backgrounds. Because glomerulonephritis was the leading cause of kidney disease in our cohort, external validity our findings is limited in populations dominated by other types of underlying kidney diseases, such as diabetes. When assessing applicability of our findings in terms of dialysis vintage, one needs to recognize that median time from beginning of dialysis to kidney transplant was 3.2 years.

## Conclusions

Randomization to kidney replacement therapy modalities is infeasible, and studies on comparative effectiveness of kidney transplant vs long-term dialysis are at high risk of bias. Our study followed target trial emulation methods to quantify life expectancy gain for kidney transplant recipients compared with patients eligible for transplant who were receiving long-term dialysis across all ages and conditional on time on wait list. We found a survival benefit for patients who receive a kidney transplant regardless of time spent on the wait list and for all ages of adults who are candidates for kidney transplant. However, data for older transplant candidates are still sparse.

## Notes

Supplement. **eMethods 1.** Target Trial Protocol **eMethods 2.** Data Management and Analysis Approach **eTable 1.** Characteristics of the Study Cohort at Treatment Allocation of Auxiliary Trials **eFigure 1.** Marginal Treatment Effect **eTable 2.** Restricted Mean Survival Time Differences for Different Ages Within for 5 and 10 Years Since Transplant Allocation **eFigure 2.** Estimated Adjusted Survival Curves for Different Ages **eFigure 3.** Estimated Adjusted Survival Curves for Different Ages Conditional on Different Times Spent Waitlisted on Dialysis **eFigure 4.** Hazard Ratios Comparing the Effect of Transplantation Versus Remaining Waitlisted on Dialysis for All-cause Mortality Across Different Ages at Time of Transplant Allocation **eFigure 5.** Hazard Ratios Comparing the Effect of Transplantation Versus Remaining Waitlisted on Dialysis for All-cause Mortality for Different Ages Conditional on Waitlisting Up to 1 Year, 1 to 2 Years, and More Than 2 Years **eFigure 6.** Distribution of Stabilized Final Weights (Product of IPTWs and IPCWs) **eTable 3.** Standardized Differences in the Stacked Data From the Auxiliary Trials Before and After Inverse Probability of Treatment Weighting **eFigure 7.** Ten-Year Restricted Mean Survival Times (RMSTs) for All-cause Mortality Estimated in Sensitivity Analyses and by the Main Analysis
eReferences

## References

[1] Yang F, Liao M, Wang P, Yang Z, Liu Y. The cost-effectiveness of kidney replacement therapy modalities: a systematic review of full economic evaluations. Appl Health Econ Health Policy. 2021;19(2):163-180. doi:10.1007/s40258-020-00614-4 33047212 PMC7902583

[2] Wong G, Howard K, Chapman JR, . Comparative survival and economic benefits of deceased donor kidney transplantation and dialysis in people with varying ages and co-morbidities. PLoS One. 2012;7(1):e29591. doi:10.1371/journal.pone.0029591 22279541 PMC3261160

[3] Haller M, Gutjahr G, Kramar R, Harnoncourt F, Oberbauer R. Cost-effectiveness analysis of renal replacement therapy in Austria. Nephrol Dial Transplant. 2011;26(9):2988-2995. doi:10.1093/ndt/gfq780 21310740

[4] Tonelli M, Wiebe N, Knoll G, . Systematic review: kidney transplantation compared with dialysis in clinically relevant outcomes. Am J Transplant. 2011;11(10):2093-2109. doi:10.1111/j.1600-6143.2011.03686.x 21883901

[5] Jensen CE, Sørensen P, Petersen KD. In Denmark kidney transplantation is more cost-effective than dialysis. Dan Med J. 2014;61(3):A4796. 24814915

[6] Hernán MA, Robins JM. Using big data to emulate a target trial when a randomized trial is not available. Am J Epidemiol. 2016;183(8):758-764. doi:10.1093/aje/kwv254 26994063 PMC4832051

[7] Glass TA, Goodman SN, Hernán MA, Samet JM. Causal inference in public health. Annu Rev Public Health. 2013;34:61-75. doi:10.1146/annurev-publhealth-031811-124606 23297653 PMC4079266

[8] Knoll GA. Is kidney transplantation for everyone? the example of the older dialysis patient. Clin J Am Soc Nephrol. 2009;4(12):2040-2044. doi:10.2215/CJN.04210609 19965539

[9] Haller MC, Kammer M, Oberbauer R. Dialysis vintage and outcomes in renal transplantation. Nephrol Dial Transplant. 2019;34(4):555-560. doi:10.1093/ndt/gfy099 29897595

[10] Hebert SA, Ibrahim HN. Kidney transplantation in septuagenarians: 70 is the new 60! Kidney Int Rep. 2019;4(5):640-642. doi:10.1016/j.ekir.2019.03.015 31080917 PMC6506756

[11] World Medical Association. World Medical Association Declaration of Helsinki: ethical principles for medical research involving human subjects. JAMA. 2013;310(20):2191-2194. doi:10.1001/jama.2013.281053 24141714

[12] von Elm E, Altman DG, Egger M, Pocock SJ, Gøtzsche PC, Vandenbroucke JP; STROBE Initiative. The Strengthening the Reporting of Observational Studies in Epidemiology (STROBE) statement: guidelines for reporting observational studies. Epidemiology. 2007;18(6):800-804. doi:10.1097/EDE.0b013e3181577654 18049194

[13] Royston P, Parmar MKB. Restricted mean survival time: an alternative to the hazard ratio for the design and analysis of randomized trials with a time-to-event outcome. BMC Med Res Methodol. 2013;13(1):152. doi:10.1186/1471-2288-13-152 24314264 PMC3922847

[14] Gran JM, Røysland K, Wolbers M, . A sequential Cox approach for estimating the causal effect of treatment in the presence of time-dependent confounding applied to data from the Swiss HIV Cohort Study. Stat Med. 2010;29(26):2757-2768. doi:10.1002/sim.4048 20803557

[15] Danaei G, Rodríguez LA, Cantero OF, Logan R, Hernán MA. Observational data for comparative effectiveness research: an emulation of randomised trials of statins and primary prevention of coronary heart disease. Stat Methods Med Res. 2013;22(1):70-96. doi:10.1177/0962280211403603 22016461 PMC3613145

[16] Robins JM, Finkelstein DM. Correcting for noncompliance and dependent censoring in an AIDS Clinical Trial with inverse probability of censoring weighted (IPCW) log-rank tests. Biometrics. 2000;56(3):779-788. doi:10.1111/j.0006-341X.2000.00779.x 10985216

[17] Glymour MM, Weuve J, Chen JT. Methodological challenges in causal research on racial and ethnic patterns of cognitive trajectories: measurement, selection, and bias. Neuropsychol Rev. 2008;18(3):194-213. doi:10.1007/s11065-008-9066-x 18819008 PMC3640811

[18] Perperoglou A, Sauerbrei W, Abrahamowicz M, Schmid M. A review of spline function procedures in R. BMC Med Res Methodol. 2019;19(1):46. doi:10.1186/s12874-019-0666-3 30841848 PMC6402144

[19] Gerhard T, Delaney JA, Cooper-Dehoff RM, . Comparing marginal structural models to standard methods for estimating treatment effects of antihypertensive combination therapy. BMC Med Res Methodol. 2012;12:119. Published online August 6, 2012. doi:10.1186/1471-2288-12-119 22866767 PMC3573973

[20] Sørensen VR, Heaf J, Wehberg S, Sørensen SS. Survival benefit in renal transplantation despite high comorbidity. Transplantation. 2016;100(10):2160-2167. doi:10.1097/TP.0000000000001002 26599492 PMC5120769

[21] Zhang Y, Gerdtham U-G, Rydell H, Jarl J. Quantifying the treatment effect of kidney transplantation relative to dialysis on survival time: new results based on propensity score weighting and longitudinal observational data from Sweden. Int J Environ Res Public Health. 2020;17(19):7318. doi:10.3390/ijerph17197318 33036407 PMC7578980

[22] Hernán MA, Sauer BC, Hernández-Díaz S, Platt R, Shrier I. Specifying a target trial prevents immortal time bias and other self-inflicted injuries in observational analyses. J Clin Epidemiol. 2016;79:70-75. doi:10.1016/j.jclinepi.2016.04.014 27237061 PMC5124536

[23] Lenain R, Boucquemont J, Leffondré K, . Clinical trial emulation by matching time-dependent propensity scores: the example of estimating impact of kidney transplantation. Epidemiology. 2021;32(2):220-229. doi:10.1097/EDE.0000000000001308 33284166

[24] Haller MC, Kainz A, Baer H, Oberbauer R. Dialysis vintage and outcomes after kidney transplantation: a retrospective cohort study. Clin J Am Soc Nephrol. 2017;12(1):122-130. doi:10.2215/CJN.04120416 27895135 PMC5220655

[25] Meier-Kriesche HU, Port FK, Ojo AO, . Effect of waiting time on renal transplant outcome. Kidney Int. 2000;58(3):1311-1317. doi:10.1046/j.1523-1755.2000.00287.x 10972695

[26] Rose C, Gill J, Gill JS. Association of kidney transplantation with survival in patients with long dialysis exposure. Clin J Am Soc Nephrol. 2017;12(12):2024-2031. doi:10.2215/CJN.06100617 29074817 PMC5718274

