# Supplementary material for: Survival Benefit of First Single-Organ Deceased Donor Kidney Transplantation Compared With Long-term Dialysis Across Ages in Transplant-Eligible Patients With Kidney Failure
Source: JAMA Netw Open. 2022 Oct 7;5(10):e2234971. doi: 10.1001/jamanetworkopen.2022.34971 (PMC9547326; doi:10.1001/jamanetworkopen.2022.34971)
Supplement: Supplement. — eMethods 1. Target Trial Protocol eMethods 2. Data Management and Analysis Approach eTable 1. Characteristics of the Study Cohort at Treatment Allocation of Auxiliary Trials eFigure 1. Marginal Treatment Effect eTable 2. Restricted Mean Survival Time Differences for Different Ages Within for 5 and 10 Years Since Transplant Allocation eFigure 2. Estimated Adjusted Survival Curves for Different Ages eFigure 3. Estimated Adjusted Survival Curves for Different Ages Conditional on Different Times Spent Waitlisted on Dialysis eFigure 4. Hazard Ratios Comparing the Effect of Transplantation Versus Remaining Waitlisted on Dialysis for All-cause Mortality Across Different Ages at Time of Transplant Allocation eFigure 5. Hazard Ratios Comparing the Effect of Transplantation Versus Remaining Waitlisted on Dialysis for All-cause Mortality for Different Ages Conditional on Waitlisting Up to 1 Year, 1 to 2 Years, and More Than 2 Years eFigure 6. Distribution of Stabilized Final Weights (Product of IPTWs and IPCWs) eTable 3. Standardized Differences in the Stacked Data From the Auxiliary Trials Before and After Inverse Probability of Treatment Weighting eFigure 7. Ten-Year Restricted Mean Survival Times (RMSTs) for All-cause Mortality Estimated in Sensitivity Analyses and by the Main Analysis eReferences [file jamanetwopen-e2234971-s001.pdf]

Supplementary Online Content

Strohmaier S, Wallisch C, Kammer M, et al. Survival benefit of first single-organ deceased donor kidney transplantation compared with long-term dialysis across ages in transplant-eligible patients with kidney failure. *JAMA Netw Open*. 2022;5(10):e2234971. doi:10.1001/jamanetworkopen.2022.34971

eMethods 1. Target Trial Protocol

eMethods 2. Data Management and Analysis Approach

eTable 1. Characteristics of the Study Cohort at Treatment Allocation of Auxiliary Trials

eFigure 1. Marginal Treatment Effect

eTable 2. Restricted Mean Survival Time Differences for Different Ages Within for 5 and 10 Years Since Transplant Allocation

eFigure 2. Estimated Adjusted Survival Curves for Different Ages

eFigure 3. Estimated Adjusted Survival Curves for Different Ages Conditional on Different Times Spent Waitlisted on Dialysis

eFigure 4. Hazard Ratios Comparing the Effect of Transplantation Versus Remaining Waitlisted on Dialysis for All-cause Mortality Across Different Ages at Time of Transplant Allocation

eFigure 5. Hazard Ratios Comparing the Effect of Transplantation Versus Remaining Waitlisted on Dialysis for All-cause Mortality for Different Ages Conditional on Waitlisting Up to 1 Year, 1 to 2 Years, and More Than 2 Years

eFigure 6. Distribution of Stabilized Final Weights (Product of IPTWs and IPCWs)

eTable 3. Standardized Differences in the Stacked Data From the Auxiliary Trials Before and After Inverse Probability of Treatment Weighting

eFigure 7. Ten-Year Restricted Mean Survival Times (RMSTs) for All-cause Mortality Estimated in Sensitivity Analyses and by the Main Analysis

eReferences

This supplementary material has been provided by the authors to give readers additional information about their work.

© 2022 Strohmaier S et al. *JAMA Network Open.*

eMethods 1: Target trial protocol

Target estimands:

- Primary: The effect of kidney transplantation compared to remaining on dialysis on time from treatment allocation to death from any cause among transplant eligible patients across different ages, quantified in terms of restricted mean survival time (RMST) differences and additionally in terms of hazard ratios and survival curves are reported.
- Secondary: The effect of kidney transplantation compared to remaining on dialysis on time from treatment allocation to death from any cause among transplant eligible patients across different ages, conditional on time spent waitlisted before treatment allocation, quantified in terms of restricted mean survival time (RMST) differences. Again, hazard ratios and survival curves are reported.

Eligibility criteria:

Inclusion

- Patients who started receiving dialysis between 1st January 2000 and 31st December 2018 and were waitlisted for their first kidney transplant between 1st January 2000 and 30th June 2019 for whom (ideal) organs from non-living donors are identified and available
- 18 years or older at the time of first waitlisting
- Registered for a single organ transplantation

Exclusion

- Patients with "highly urgent" initial waitlisting status
- Patients who had previously received a kidney transplant

Treatment strategies

- Receive the transplant (from a non-living donor) immediately (treatment group TX=1) or
- Do not receive the transplant and never receive a transplant in the future (remaining on dialysis) (control group TX=0)

Assignment procedures

- Randomization to treatment or control group takes place at the time a donor organ becomes available.
- Non-blinded

Outcome

- Time to death from any cause. Follow-up time starts at the time an organ for transplantation becomes available and patients are observed until loss-to-follow-up, death or end of the observation period on 30th June 2019.

© 2022 Strohmaier S et al. *JAMA Network Open.*

eMethods 2: Data management and analysis approach

Data management and handling of missing data

Figure 1 in the main text gives an overview of the number of excluded patients and reasons why they were removed from the study sample.

The final sample included 4,445 patients with a total of 15,755 observations. The table below gives the number and percentage of missing values before imputation.

Given the relatively small percentage of missing values we decided to apply simple imputation techniques. Missing values in comorbidities and number of blood pressure lowering drugs were imputed by carrying the last observation forward. For comorbidities one can argue that once they are present, the fact that an individual experienced that particular comorbidity remains unchanged in the future. For body mass index (BMI), first low values for height and weight were winsorized at the 0.5th percentile, then BMI was calculated as weight/(height/100)^2 and truncated at 15 and 45 kg/m². Missing BMI values were imputed by carrying the last observation forward. If the value was missing at first waitlisting, we applied last observation carried backward.

**Patient characteristics**	**Missing values before imputation**
Height in cm	191 (1.2)
Weight in kg	116 (0.7)
BMI in kg/m²	257 (1.6)
Number of blood pressure lowering drugs	111 (0.7)
Diabetes mellitus	83 (0.5)
Coronary heart disease	91 (0.6)
Myocardial infarction or instable angina pectoris	91 (0.6)
Congestive heart insufficiency	91 (0.6)
Other heart disease	91 (0.6)
Neoplasia	94 (0.6)
Liver disease	92 (0.6)
Cerebrovascular disease	89 (0.6)
Peripheral vascular disease	89 (0.6)

Abbreviations: BMI, body mass index.

© 2022 Strohmaier S et al. *JAMA Network Open.*

Specific analysis steps

To estimate the target estimands we performed the following analyses steps:

1) Create a series of auxiliary trials – one at each transplant allocation time
2) "Stack" the data from all auxiliary trials
3) Estimate stabilized inverse probability of treatment weights (IPTW)
4) Estimate stabilized inverse probability of censoring weights (IPCW)
5) Fit weighted survival models and calculate effect estimates on different outcome scales

The details of each step are given below.

Step 1: Create a series of auxiliary trials

Patients were included in the study at the time they were waitlisted which marks time zero of the individual. Let further be $TX_i(t)$ the treatment (transplant) indicator function for the i-th individual at time t after initial waitlisting.

Let $s_1, \dots, s_K$ denote the distinct time points counted in years since being waitlisted where one or multiple patients received a transplant. At each of these transplantation times $s_1, \dots, s_K$ an auxiliary trial was initiated. In the k-th auxiliary trial patients with active waitlisting status were included who had survived for at least s_k years since being waitlisted.

For the k-th trial starting at time s_k, data were arranged as follows:

- All patients transplanted at time s_k after their first waitlisting (often only one patient) were assigned to the treatment group (variable $TX = 1$) if they had a non-living donor and did not undergo a "high urgency" transplantation.
- All other transplantable patients with active waitlisting status at time s_k and under observation were eligible for the control group ($TX = 0$). Controls who were transplanted at later times were artificially censored and consequently assigned to the treated group in a later trial of the series. Patients who were set to "high urgency" during follow-up of an auxiliary trial were censored and not considered for a new auxiliary trial.
- Covariates $L(s_k)$ from the closest recording before s_k were included.

The following figure gives an overview of the number of patients per auxiliary trial:

© 2022 Strohmaier S et al. *JAMA Network Open.*

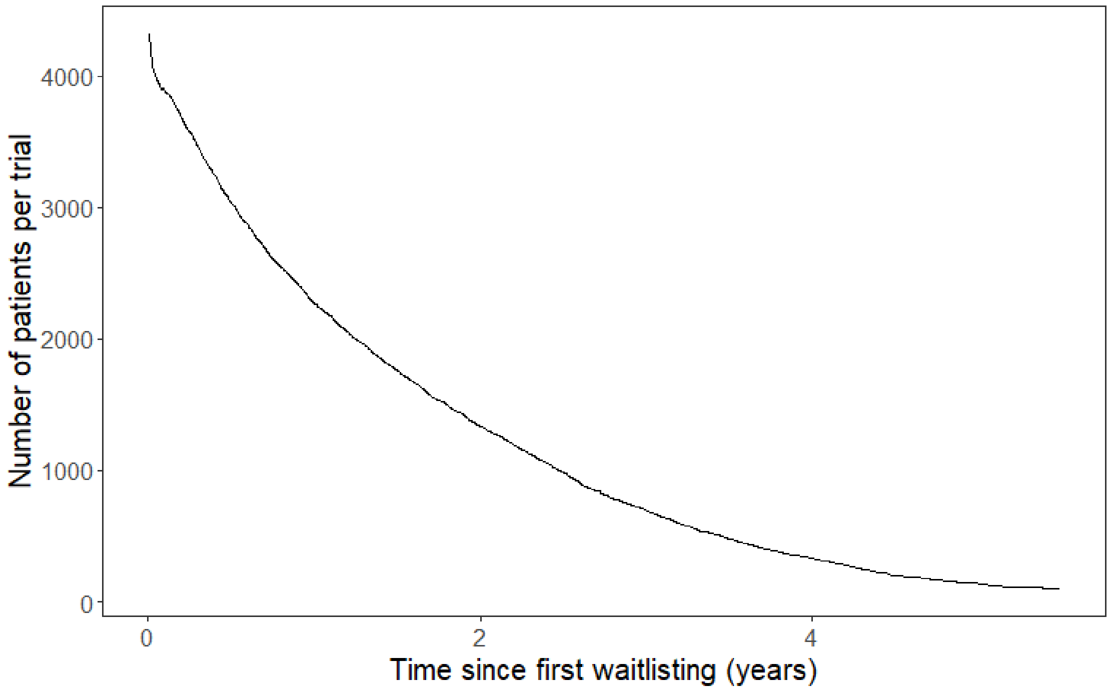

In the analysis, we only included trials with $s_k \leq 5.5$ years, i.e. we only considered transplantations performed within 5.5 years since time of first waitlisting. This cut-off results in 1367 trials with at least 101 patients per trial, thereby mitigating issues with model fit. Slightly abusing notation, we will in the following use K for the number of trials with $s_k \leq 5.5$ years.

Step 2: "Stack" the data from all auxiliary trials

All auxiliary trials were stacked into one data set.

Step 3: Estimate stabilized inverse probability of treatment weights (IPTWs)

To mimic random allocation for each of the K auxiliary trials we created a pseudo population using stabilized inverse probability of treatment weights (IPTWs). First, to obtain unstabilized IPTWs $w_i^{TX}(k)$ for the i-th patient in the k-th trial, we had to estimate each patient's inverse probability to initiate treatment at time point s_k using covariates fixed at first waitlisting time and time-varying covariates at s_k (denominator model). Further, to stabilize the weights $w_i^{TX}(k)$ we multiplied them by the probabilities obtained from fitting an additional (numerator) model only including an intercept, resulting in the stabilized weights $sw_i^{TX}(k)$. Once patients were transplanted they remained in the treatment group.

For the denominator model, we fit a pooled logistic regression model to the stacked data set where each patient could contribute several rows, one for each trial. The pooled logistic regression model was defined as $P(TX(s_k) = 1 \,|\mathrm{L}(s_k)) = \mathrm{expit}(a + \alpha_0(s_k) + \alpha_1 \mathrm{L}(s_k))$ where a denotes a global intercept, $\alpha_0(s_k)$ is a time-dependent intercept and $\mathrm{L}(s_k)$ are covariates from the closest recording and $\mathrm{expit}(x) = e^x/(1 + e^x)$. $\mathrm{L}(s_k)$ consisted of age, sex, the year of first waitlisting, dialysis before waitlisting (yes/no) and at all trial initiation times the most recent values of BMI, comorbidities (diabetes mellitus, coronary heart disease, myocardial infarction or instable angina pectoris, congestive heart insufficiency, other heart disease, neoplasia, liver disease, cerebrovascular disease, peripheral vascular disease), number of blood pressure lowering drugs, time from waitlisting to trial start, and non-transplantable periods before trial start (yes/no). Continuous variables were modeled with restricted cubic splines, in particular, we used 5 knots (at 1%, 25%, 50%, 75% and 99% quantiles) for age and BMI and 3 knots (at 10%, 50%, and 90%

© 2022 Strohmaier S et al. *JAMA Network Open.*

quantiles) for year of first waitlisting and for number of blood pressure medication. For estimation of IPTWs, the time-dependent intercept, $\alpha_0(s_k)$ was not estimated in each trial separately but it was modelled with restricted cubic splines using 5 knots at the 1%, 25%, 50%, 75% and 99% quantiles borrowing strength between trials.
Analogously, for the numerator model we fit a pooled logistic regression model as described above, but only including an intercept.

Based on the denominator model the estimated predicted probabilities $\hat{p}_i(k) = \text{expit}(\hat{a} + \hat{\alpha}_0(s_k) + \hat{\alpha}_1 L(s_k))$ are the probability that the i-th patient received a transplant in the k-th trial. The estimate of the IPTW for the i–th patient, who does not receive a transplant in the k-th trial, is $\widehat{w}_i^{TX}(k) = \frac{1}{1-\hat{p}_i(u)}$. For the i-th patient receiving a transplant in the k-th trial, the IPTW is $\widehat{w}_i^{TX}(k) = 1/\hat{p}_i(k)$.
To obtain stabilized weights $\widehat{sw}_i^{TX}(k)$ we multiplied $\widehat{w}_i^{TX}(k)$ with the predicted probabilities from the numerator model calculated analogously as described above.

Step 4: Estimate stabilized inverse probability of censoring weights (IPCWs)

In each auxiliary trial of the series, patients in the control group were artificially censored at time of transplantation since they did not adhere to their assigned treatment strategy anymore (no transplantation now and never being transplanted in the future). To correct for this artificial censoring, we computed time-varying inverse probability of censoring weights (IPCWs) separately for each trial. IPCWs were only computed for the control group. In the treatment group, the weights were set to one.

To obtain annually varying IPCWs, we fit a Cox model for the time to censoring $T_i^{(k),C}$ in the k-th auxiliary trial.[1,2] We reshaped the data into counting process format, such that within each trial each patient contributed multiple rows corresponding to the yearly intervals with covariate information and outcome data. Only in the last interval of a patient the censoring indicator $C_i^{(k)}(t) := I\left(T_i^{(k),C} \le t\right)$ was set to 1 if the patient had been censored (i.e. censored due to loss-to-follow-up, end of study period, or receiving a transplantation), otherwise the censoring indicator was 0.

To estimate IPCWs $\widehat{w}_i^{(k),\bar{C}}(t)$ for each patient i at time t, a Cox model for each trial was defined as $\lambda^{(k)}(t) = \lambda_0^{(k)}(t)\exp(\gamma G(k))$ where $G(k)$ represents the covariate values at the start of k-th trial. We considered sex, the year of first waitlisting as well as age, BMI, comorbidities and number of blood pressure lowering drugs as covariates in the model.

For yearly intervals we extracted the patients' probability of being uncensored at the beginning of that interval given that they had not been censored in the interval before from the estimated survival curve $\hat{S}_i^k(t) = \hat{S}_0^k(t)^{\exp(\hat{\gamma}\, G(k))}$, with $\hat{S}_0^k(t) = \exp\left(-\sum_{u=0}^{t} \hat{\lambda}_0^k(u)\right)$, to obtain the IPCWs as $\widehat{w}_i^{(k),\bar{C}}(t) = 1/\hat{S}_i^k(t)$.

To stabilize the IPCW we calculated a Kaplan-Meier estimate and extracted the probabilities of being censored at time t from the survival curves and further multiplied these probabilities with $\widehat{w}_i^{(k),\bar{C}}(t)$ to obtain $\widehat{sw}_i^{(k),\bar{C}}(t)$.

© 2022 Strohmaier S et al. *JAMA Network Open.*

Step 5: Fit weighted survival models and calculate effect estimates on different outcome scales

The final time-dependent stabilized weights for the i-th patient in the k-th auxiliary trial were obtained as the products of stabilized IPTWs and stabilized yearly IPCWs with $\widehat{sw}_i(t) = \widehat{sw}_i^{TX}(k) * \widehat{sw}_i^{(k),\bar{C}}(t)$ (van der Wal and Geskus, p. 15 or Hernan & Robins, part 4, p. 23) and were winsorized at the 1st and 99th percentile.[3,4]

To obtain the marginal effect of transplantation we fit a weighted Cox proportional hazards model for all-cause mortality, using $\widehat{sw}_i(t)$ as time-dependent weights and treatment assignment as main exposure. To evaluate whether the effect of transplant was modified by age at trial initiation, we fit a model including interaction terms between the treatment variable and four restricted cubic spline bases of age to allow for flexibility.

Further, to investigate whether the effect of transplantation was dependent on the time on waitlist before trial initiation we estimated separate models for patients who had spent up to 1 year, between 1 and 2 years, or more than 2 years on the waitlist before trial initiation. In all three analyses we included interaction terms between treatment and age as described above.

Confidence intervals were calculated as the 2.5th and 97.5th percentiles from the distribution of the respective estimand estimated in 1000 bootstrap samples. If in any bootstrap sample the frequency of any category of a categorical covariate was below 5, the covariate was removed from the model fit in this sample to avoid non-convergence.

We report our results in terms of restricted mean survival time (RMST) differences and supplementary in terms of survival curves and hazard ratios. To obtain RMST difference and survival curve estimates, we plugged in every variable combination of interest into the respective model.

Assumptions

The applied causal inference methodology requires several structural assumptions:

- No unmeasured confounding - we assume that our information on measured confounders was sufficient to ensure exchangeability of our treatment groups after adjustment by means of inverse probability of treatment weights.
- Positivity - Each participant had a positive probability to receive either treatment at each transplantation allocation time. Since we only considered patients on the waitlist and hence eligible for transplantation, we ruled out any structural violation of the positivity assumption. Any sampling related violations would have been detected during the inverse probability of treatment weight calculations, e.g. by running into divergence or separation problems.
- Consistency at all transplant allocation times – If a patient indeed received a transplant at a certain time s_k, we assume that their survival is the same as it would have been had they been forced to receive a transplant at time s_k. Likewise, we assume that the survival of patients who did not receive a transplant at a certain time s_k is the same as it would have been had they been forced to deny a transplant at time s_k. As organs from non-living donors become available at essentially unpredictable times this is likely to hold. Also, the transplantation surgery itself is a standardized procedure.

© 2022 Strohmaier S et al. *JAMA Network Open.*

- No interference – as stated in the inclusion criteria in our target trial protocol we assume that for each patient the ideal organs from non-living donors are identified and available at each transplant time.

Furthermore, application of the Cox model implies the proportional hazards assumption. Regarding the outcome models in the main analyses it is well known that hazards are non-proportional shortly after transplantation because the mortality rate within the first year after transplantation is higher than for patients on dialysis. However, the main focus of our study was on long-term survival after kidney transplantation. Given the number of censoring weight models the assumption could not be checked manually for each artificial trial in the series. Manually checking a number of random trials we did not find any serious departures from the proportional hazards assumption.

Unfortunately, we did not have access to the organ allocation lists generated by Eurotransplant, which match transplant candidates to available donor organs. This introduced the implicit assumption that an ideal organ would be available for each eligible patient at each transplant allocation time. However, the lack of details on transplant offers may not be relevant, as there is evidence from the Organ Procurement and Transplantation Network database for a survival benefit of accepting the first kidney allograft offer compared to hopes of receiving a better allograft and remaining waitlisted on dialysis.[5] A caveat of this study from Cohen et al. (2019) is however that temporary transplant ineligibility was not accounted for.

© 2022 Strohmaier S et al. *JAMA Network Open.*

eTable 1: Characteristics of the study cohort at treatment allocation of auxiliary trials

This table is based on a total of 2,067,620 ‘person-trials’.

	Transplanted N=3,163	Not transplanted N=2,064,457
Women	34.2%	34.4%
Men	65.8%	65.6%
Age in years, *mean (sd)*	54.8 (12.8)	52.5 (12.9)
BMI in kg/m², *mean (sd)*	26.6 (4.8)	26.2 (4.6)
Renal disease		
Diabetic nephropathy	16.6%	15.1%
Glomerulonephritis	27.7%	29.2%
Hereditary	17.0%	17.4%
Vascular	15.9%	14.9%
Others	22.8%	23.4%
Dialysis before waitlisting	96.4%	94.0%
If so, time on dialysis before waitlisting in days, *mean (sd)*	511.6 (546.8)	362.5 (358.6)
Number of antihypertensives, *mean (sd)*	1.5 (1.6)	1.5 (1.6)
Diabetes mellitus	24.9%	21.3%
Coronary heart disease	26.6%	20.7%
Myocardial infarction or instable angina pectoris	5.3%	4.1%
Congestive heart insufficiency	10.0%	8.2%
Other heart disease	21.2%	16.6%
History of neoplasia	9.5%	6.5%
Liver disease	11.4%	9.5%
Cerebrovascular disease	15.4%	11.7%
Peripheral vascular disease	18.5%	15.1%

Abbreviation: BMI, body mass index.

© 2022 Strohmaier S et al. *JAMA Network Open.*

eFigure 1: Marginal treatment effect

(A) Marginal treatment effect adjusted for time-dependent confounding and artificial censoring expressed as restricted mean survival times (RMST) and their difference

In the left figure, the RMST depending on the length of follow-up is given for transplanted (red) and non-transplanted patients (blue). The right figure shows the difference in the RMST. 95% confidence intervals are shaded.

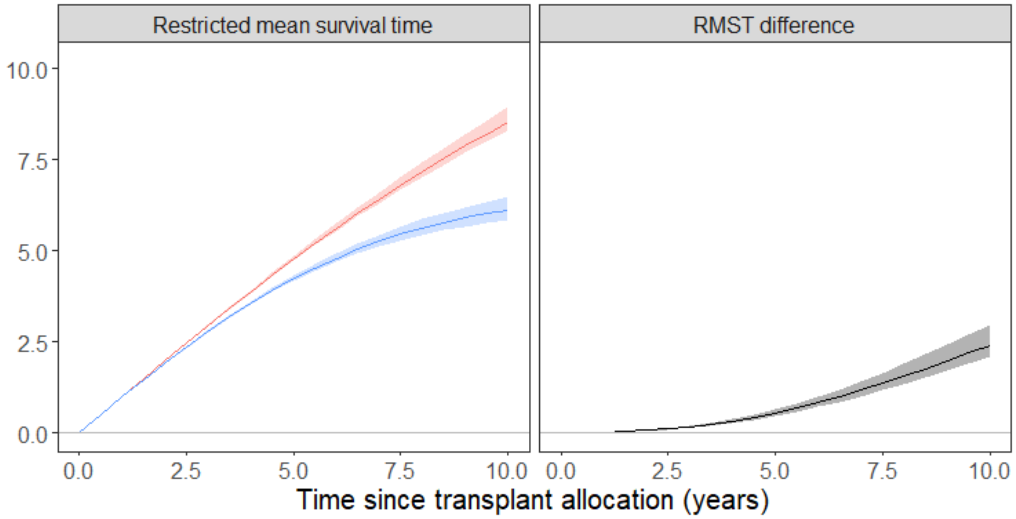

(B) Marginal treatment effect adjusted for time-dependent confounding and artificial censoring expressed as survival probabilities and their difference

In the left figure, survival probabilities are given for transplanted (red) and non-transplanted patients (blue). The right figure shows the difference in the survival probability. 95% confidence intervals are shaded.

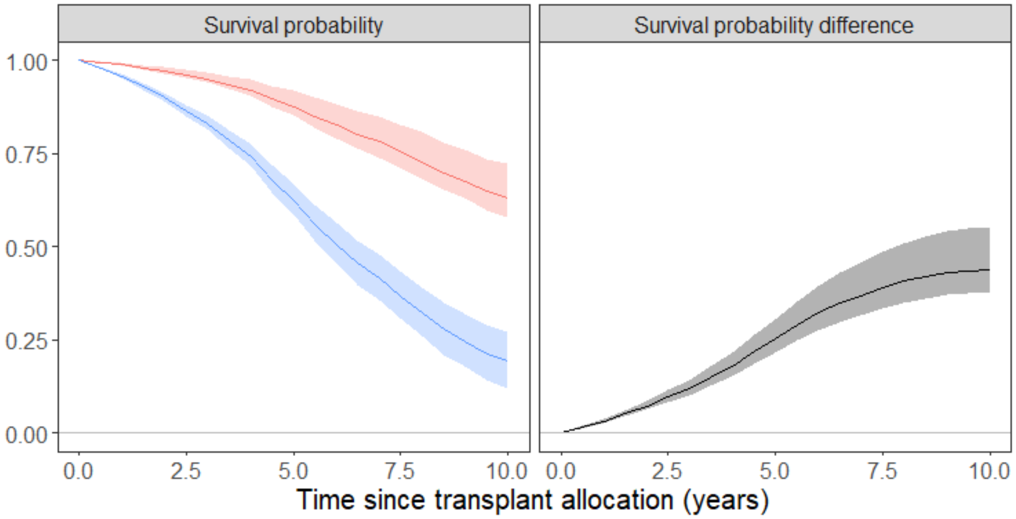

© 2022 Strohmaier S et al. *JAMA Network Open.*

eTable 2: Restricted mean survival time differences for different ages within for 5 and 10 years since transplant allocation

	5 years since transplant allocation			10 years since transplant allocation		
Age	RMST differences	95% confidence interval		RMST differences	95% confidence interval	
20	0.10	-0.02	0.33	0.57	-0.14	1.84
30	0.15	0.05	0.27	0.84	0.27	1.47
40	0.24	0.12	0.36	1.27	0.70	1.94
50	0.41	0.31	0.52	2.03	1.56	2.55
60	0.77	0.63	0.91	3.01	2.50	3.54
70	0.79	0.58	0.99	2.48	1.88	3.04

Abbreviation: RMST, restricted mean survival time

© 2022 Strohmaier S et al. *JAMA Network Open.*

eFigure 2: Estimated adjusted survival curves for different ages

Survival probabilities are given for transplanted (red) and non-transplanted patients (blue) for different transplant candidate ages. 95% confidence intervals are shaded.

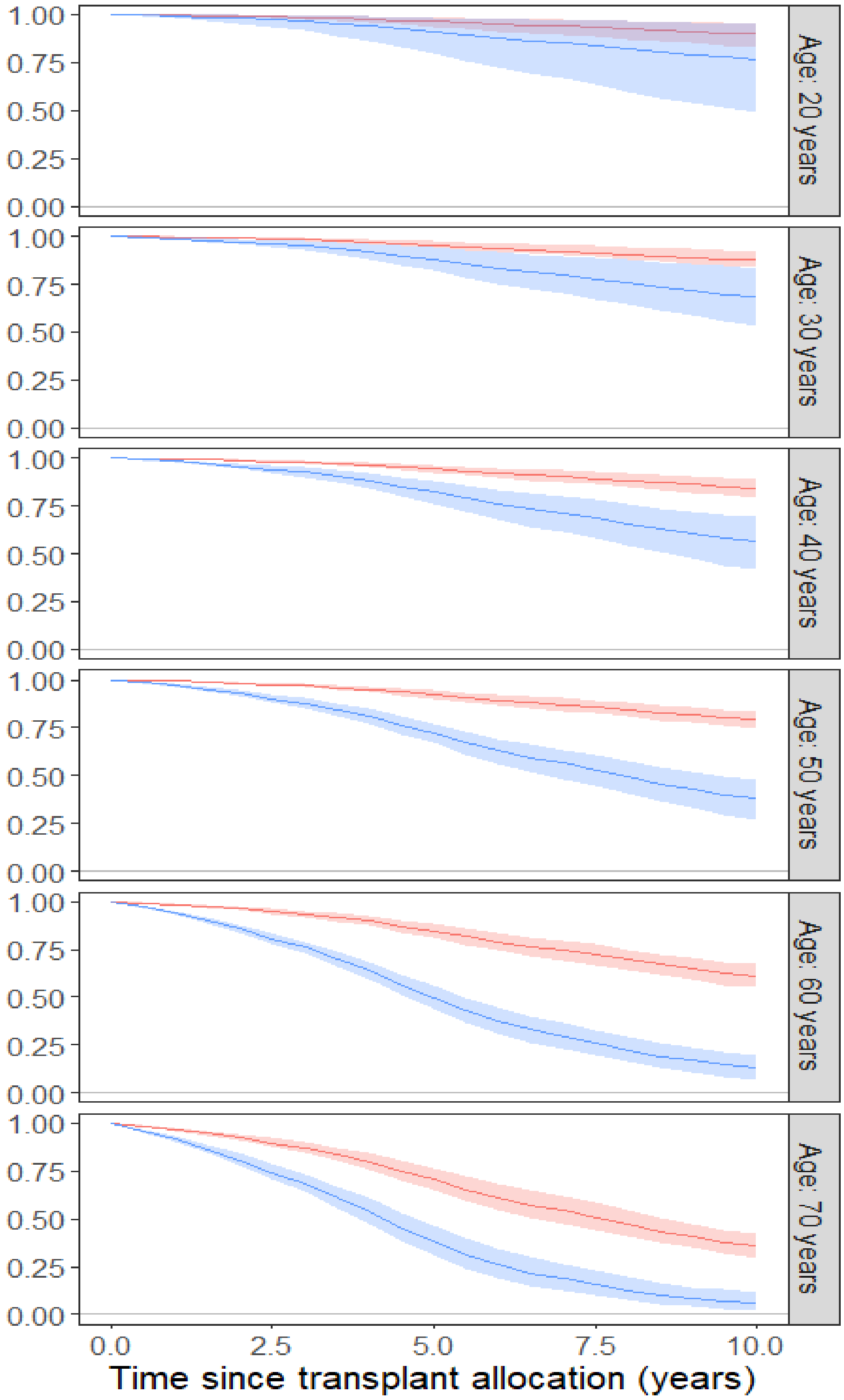

© 2022 Strohmaier S et al. *JAMA Network Open.*

eFigure 3: Estimated adjusted survival curves for different ages conditional on different times spent waitlisted on dialysis

Survival probabilities are given for transplanted (red) and non-transplanted (blue) patients for different ages waitlisted up to 1 year, 1 to 2 years and more than 2 years. 95% confidence intervals are shaded. Note that by definition of the exclusion criteria all transplanted patients aged 20 years or younger were waitlisted less than 2 years.

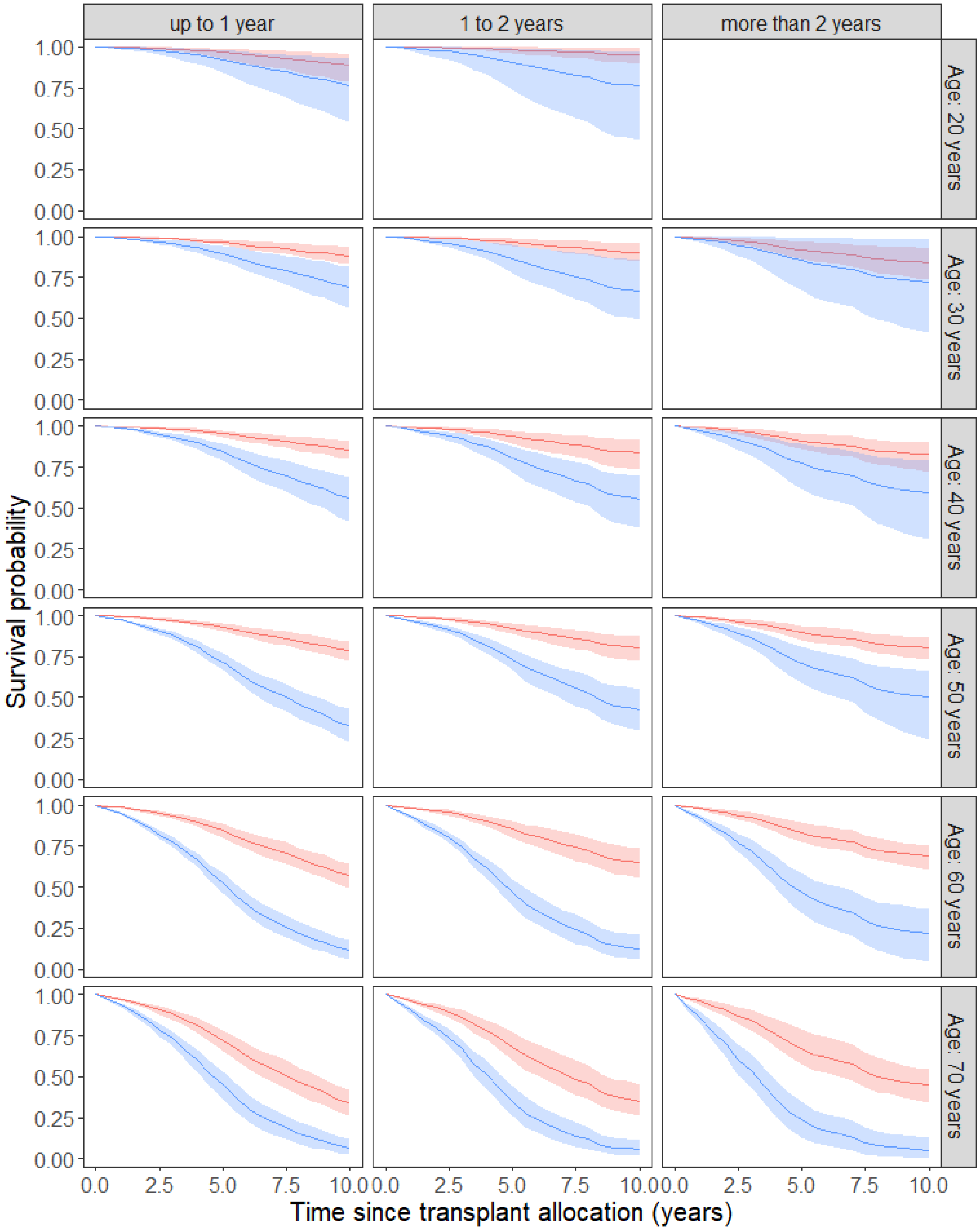

© 2022 Strohmaier S et al. *JAMA Network Open.*

eFigure 4: Hazard ratios comparing the effect of transplantation versus remaining waitlisted on dialysis for all-cause mortality across different ages at time of transplant allocation.

95% confidence intervals are shaded.

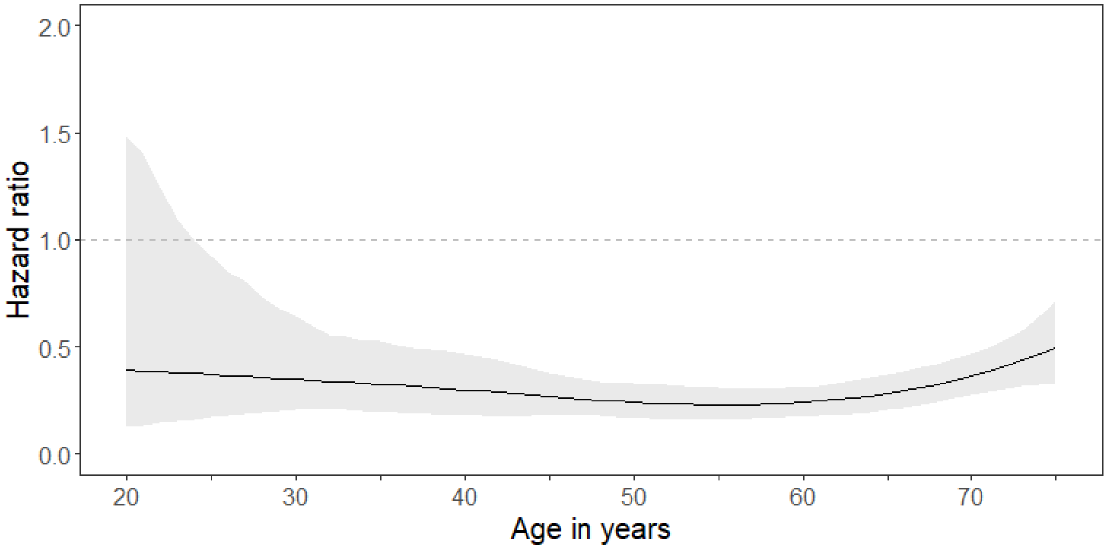

© 2022 Strohmaier S et al. *JAMA Network Open.*

eFigure 5: Hazard ratios comparing the effect of transplantation versus remaining waitlisted on dialysis for all-cause mortality for different ages conditional on waitlisting up to 1 year, 1 to 2 years and more than 2 years.

95% confidence intervals are shaded.

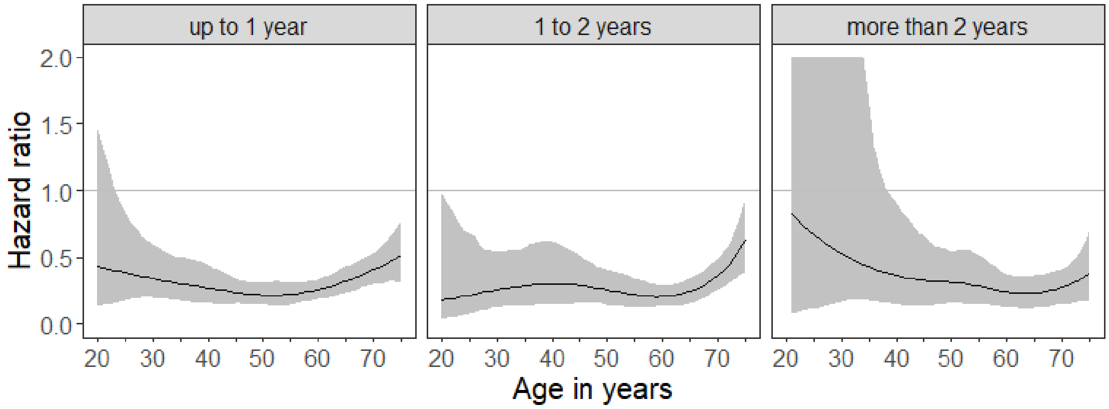

© 2022 Strohmaier S et al. *JAMA Network Open.*

eFigure 6: Distribution of stabilized final weights (product of IPTWs and IPCWs)

Weights winsorized at the 0.1st and 99.9th percentile of their distribution, used for the main analysis presented in the manuscript. The median (interquartile range) of the stabilized weights was 0.68 (0.60, 0.79).

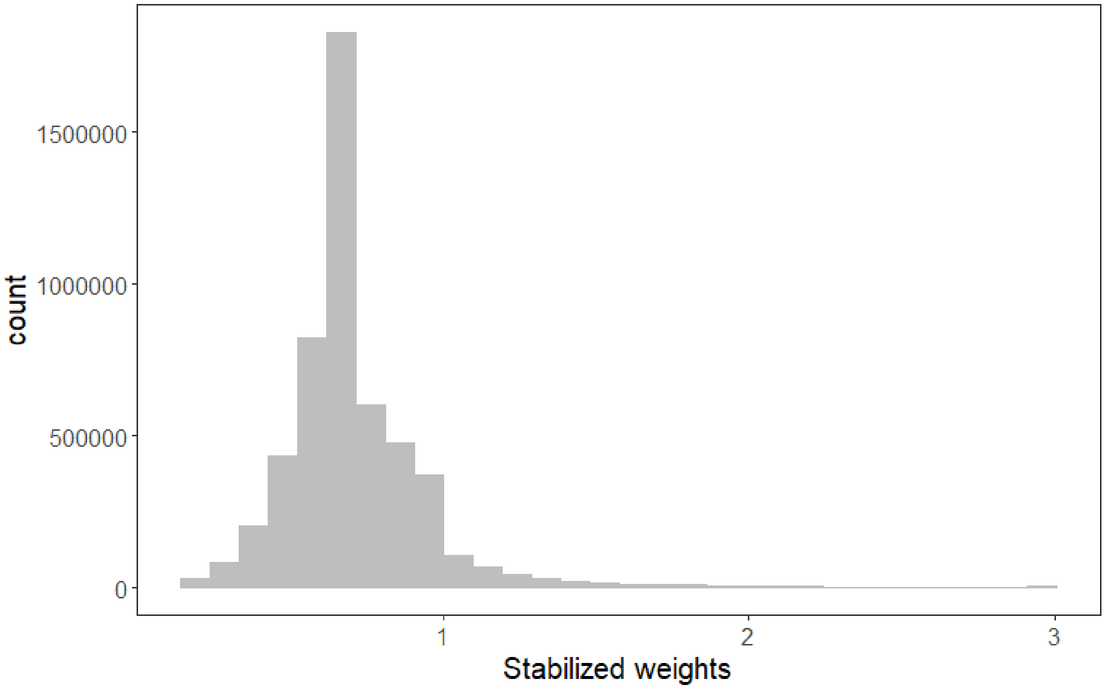

© 2022 Strohmaier S et al. *JAMA Network Open.*

eTable 3: Standardized differences in the stacked data from the auxiliary trials before and after inverse probability of treatment weighting

	Standardized differences	
	before weighting	after weighting
Sex	-0.003	0.013
Age in years	0.182	-0.008
BMI	0.090	-0.021
Dialysis before waitlisting (yes/no)	0.112	0.018
Number of antihypertensives	0.011	-0.011
Diabetes mellitus	0.086	0.005
Coronary heart disease	0.138	-0.001
Myocardial infarction or instable angina pectoris	0.056	0.020
Congestive heart insufficiency	0.062	0.027
Other heart disease	0.117	0.011
History of neoplasia	0.111	0.011
Liver disease	0.063	0.016
Cerebrovascular disease	0.109	0.005
Peripheral vascular disease	0.091	0.027
Year of first waitlisting	0.213	-0.016
Time from waitlisting to trial start	0.384	0.013
Non-transplantable periods before trial start (yes/no)	0.398	-0.010

Abbreviation: BMI, body mass index.

© 2022 Strohmaier S et al. *JAMA Network Open.*

eFigure 7: Ten-year restricted mean survival times (RMSTs) for all-cause mortality estimated in sensitivity analyses and by the main analysis

The plots show 10 year RMST for all-cause mortality in years for transplanted group in red and for the control group (remaining waitlisted on dialysis) in blue across considered candidate ages (x axis). Solid lines are the estimates from three sensitivity analyses, dashed lines are the estimates from the main analysis as presented in Figure 2. Sensitivity analysis 1 winsorizes the final weights at the 1st and 99th percentile instead of the 0.1st and 99.9th percentile as the main analysis. Sensitivity analysis 2 uses unstabilized inverse probability of treatment weights and inverse probability of censoring weights. In sensitivity analysis 3, the inverse probability of censoring weights are calculated for 6 months intervals instead of annual intervals.

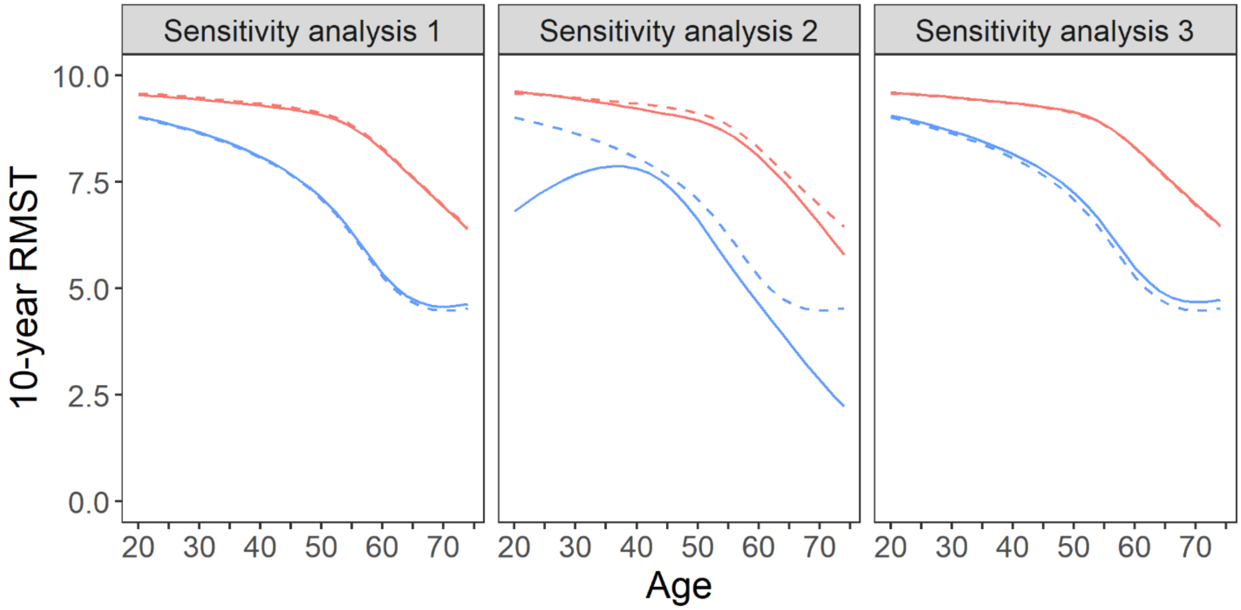

© 2022 Strohmaier S et al. *JAMA Network Open.*

eReferences

1. Robins JM, Finkelstein DM. Correcting for noncompliance and dependent censoring in an AIDS clinical trial with inverse probability of censoring weighted (IPCW) log-rank tests. *Biometrics.* 2000;56(3):779-788. doi:10.1111/j.0006-341X.2000.00779.x
2. Graffeo N, Latouche A, Le Tourneau C, Chevret S. ipcwswitch: An R package for inverse probability of censoring weighting with an application to switches in clinical trials. *Comput Biol Med.* 2019;111:103339. doi:10.1016/j.compbiomed.2019.103339
3. Hernán M, Robins J. *Causal Inference: What If.* Boca Raton: Chapman & Hall/CRC; 2020.
4. van der Wal WM, Geskus RB. ipw: An R Package for Inverse Probability Weighting. *Journal of Statistical Software; Vol 1, Issue 13 (2011).* 2011. doi:10.18637/jss.v043.i13
5. Cohen JB, Potluri V, Porrett PM, et al. Leveraging marginal structural modeling with Cox regression to assess the survival benefit of accepting vs declining kidney allograft offers. *Am J Transplant.* 2019;19(7):1999-2008. doi:10.1111/ajt.15290

© 2022 Strohmaier S et al. *JAMA Network Open.*
